# Partially purified components of *Uncaria sinensis* attenuate blood brain barrier disruption after ischemic brain injury in mice

**DOI:** 10.1186/s12906-015-0678-4

**Published:** 2015-05-27

**Authors:** Hyung Bum Seo, Bo Kyung Kang, Ji Hyun Kim, Young Whan Choi, Jin Woo Hong, Byung Tae Choi, Hwa Kyoung Shin

**Affiliations:** Division of Meridian and Structural Medicine, School of Korean Medicine, Pusan National University, Yangsan, Gyeongnam 626-870 Republic of Korea; Department of Horticultural Bioscience, College of Natural Resource and Life Science, Pusan National University, Miryang, Gyeongnam 626-706 Republic of Korea; Division of Clinical Medicine 1, School of Korean Medicine, Pusan National University, Yangsan, Gyeongnam 626-870 Republic of Korea; Korean Medical Science Research Center for Healthy-Aging, Pusan National University, Yangsan, Gyeongnam 626-870 Republic of Korea

**Keywords:** Blood–brain barrier, Focal cerebral ischemia, MMP, Tight junction protein, *Uncaria sinensis*

## Abstract

**Background:**

*Uncaria sinensis* (US) has long been used in traditional Korean medicine to relieve various nervous-related symptoms and cardiovascular disease. We recently showed the neuroprotective and cerebrovascular protective effects of US on cerebral ischemia; however, its effects on the blood–brain barrier (BBB) are poorly understood. In this study, the effects of partially purified components of US (PPUS) on BBB disruption were investigated in mice subjected to ischemic brain injury.

**Methods:**

Focal cerebral ischemia was induced in C57BL/6J mice by photothrombotic cortical ischemia. PPUS was injected intraperitoneally 30 min before ischemic insults. Infarct volume, neurological score, wire-grip test, Evans blue leakage and brain water content were then examined 24 h after ischemic brain injury.

**Results:**

Infarct volume was significantly reduced and neurological deficit and motor deficit were greatly improved in PPUS-pretreated mice relative to those treated with vehicle following photothrombotic cortical ischemia. Brain edema-induced change of Evans blue extravasation and water content in the ipsilateral hemisphere were alleviated by treatment with PPUS. In addition, PPUS significantly reduced ischemic brain injury-induced degradation of tight junction proteins and elevation of matrix metalloproteinase-9 (MMP-9).

**Conclusions:**

PPUS prevents cerebral ischemic damage by BBB protection, and these effects were associated with inhibition of tight junction degradation and MMP-9 induction.

## Background

Stroke is the third leading cause of death and the main cause of adult disability worldwide [1]. Despite numerous researches and active challenges, the treatment of acute stroke remains limited. In addition, most therapeutic approaches developed in the laboratory have focused on protecting neurons, but these developed treatments have often failed in the clinical trials [2]. Currently, limited advances have been made in developing therapies to reduce the deleterious effects of ischemic stroke, whereas efforts in prevention have reduced stroke incidence and mortality [3]. Therefore, considerable interest has been increased in stroke prevention, particularly the use of natural medicines.

The blood–brain barrier (BBB), which is primarily formed by specialized brain endothelial cells interconnected by well-developed tight junctions, provides a dynamic interface between the brain and blood circulation that maintains central nervous system homeostasis [4]. During the acute phase of ischemic stroke, the BBB is the first structure to be injured, and stroke-induced BBB disruption can lead to further progression of brain damage [5]. Degradation of the cerebrovascular ZO-1 and claudin as well as the matrix metalloproteinase (MMP) has been shown to be highly correlated with the dynamic process of BBB disruption after cerebral ischemia [6, 7]. Among MMPs, MMP-9 has been most intensively studied for its involvement in BBB disruption after stroke [7]. Thus, protecting the BBB and maintaining its integrity might be a beneficial method of alleviating brain damage. In addition, development of drugs to prevent BBB disruption has become a popular topic in the pharmaceutical community.

Seeking pharmacodynamic natural medicines is currently gaining popularity in pharmaceutical development. The treatment of ischemic brain disease by natural medicines has a long history, and has accumulated a rich theoretical knowledge and treatment experience. The hook and stems of *Uncaria sinensis* (Oliv.) Havil (US) are regarded as the main active ingredient comprising Choto-san (“Chotoko” in Chinese), which is used for treatment of cardiovascular disease and various nervous-related symptoms [8]. There is ample evidence of the beneficial neuroprotective effects of US based on both in vivo and in vitro studies. US exerts neuroprotective effects against glutamate-induced neuronal damage [9, 10] and transient forebrain ischemic injury [11, 12]. More recently, our group demonstrated that US exerted cerebrovascular protective action through an eNOS-dependent mechanism in cerebral ischemic mice and anti-apoptotic properties against glutamate-induced neurotoxicity in primary cultured cortical neurons [13, 14]. Based on these findings, we hypothesized that US exerts beneficial effects against ischemic brain damage by affecting the structure and function of the BBB. Therefore, in this study, we investigated the protective effects of partially purified US components (PPUS) against ischemic brain injury and its molecular mechanisms in a mouse model of focal cerebral ischemia. To accomplish this, we examined whether preischemic PPUS treatment ameliorates photothrombotic cortical ischemia-induced BBB disruption and brain edema and, if so, whether these protective effects are associated with inhibition of tight junction protein degradation and MMP-9 elevation in the brain.

## Methods

### Preparation of PPUS extracts

Dried hooks and stems of US were purchased from Hwalim Natural Drug (Busan, Korea) in September 2010 and authenticated by one of the study authors (Jin Woo Hong). A voucher specimen (accession number PDRLCW-1) has been deposited in the Plant Drug Research Laboratory of Pusan National University (Mirang, Korea). The dried hooks and stems of US (2.0 kg) were ground to a fine powder and then successively extracted at room temperature with *n*-hexane, ethyl acetate and methanol. Briefly, hexane extracts of US were filtered and evaporated under reduced pressure at 45 °C and then lyophilized, which yielded a white powder of hexane extract (14.54 g). The hexane extract (11.31 g) was subjected to chromatography on a silica gel (40 μm; J.T. Baker, Phillipsburg, NJ, USA) column (70 x 8.0 cm) with a step gradient of 50 % CHCl_2_ in hexane, 100 % CHCl_2_, 5 and 20 % acetone in CHCl_2_ and 5, 25 and 50 % MeOH in CHCl_3_ to obtain 62 fractions. Of these extracts, the solid form of fraction 43 (23.1 mg) was dissolved with dimethyl sulfoxide (DMSO) for further experiments.

### Focal cerebral ischemia

Male C57BL/6J mice (DooYeol Biotech, Seoul, Korea) weighing 20–25 g were housed under diurnal lighting conditions and allowed food and tap water ad libitum. This study was carried out in strict accordance with the recommendations in the Guide for the Care and Use of Laboratory Animals of the National Institutes of Health. In addition, the animal protocol used in this study has been reviewed by the Pusan National University - Institutional Animal Care and Use Committee (PNU-IACUC) on their ethical procedures and scientific care, and it has been approved (Approval Number: PNU-2011-000367). Computer-generated randomization was conducted by SigmaPlot 11.2 (Systat Software Inc, San Jose, CA) for allocating to vehicle group, 1 mg/kg PPUS-treated group or 3 mg/kg PPUS-treated group. After getting the random number by computer-generated randomization, C57/BL6J male mice were allocated in a blinded fashion. Focal cerebral ischemia was induced by photothrombosis of the cortical microvessels [15]. Anesthesia was achieved by isoflurane (2 % induction and 1.5 % maintenance, in 80 % N_2_O and 20 % O_2_) administered via a face mask in a stereotaxic frame (David Kopf Instruments, Tujunga, CA). The depth of anesthesia was checked by the absence of cardiovascular changes in response to a tail pinch. Rectal temperature was maintained at 36.5–37.5 °C using a Panlab thermostatically controlled heating mat (Harvard Apparatus, Holliston, MA). Rose Bengal (Sigma-Aldrich, St. Louis, MO), 0.1 ml of a 10 mg/ml solution in sterile saline, was injected intraperitoneally (ip) 5 min before illumination. The midline of the scalp was incised, pericranial tissues were dissected, and the bregma and lambda points were identified. For illumination, a fiber optic bundle of a KL1500 LCD cold light source (Carl Zeiss, Jena, Germany) with a 4 mm aperture was centered 2.4 mm laterally from the bregma using a micromanipulator, where the mouse sensorimotor cortex is located. The brain was illuminated through the exposed intact skull for 15 min. Finally, the surgical wound was sutured and the mice were allowed to recover from anesthesia. The control mice were done the same procedure except the cold light illumination.

### Infarct volumes and edema

Mice were deeply anesthetized with thiopental sodium 24 h after ischemic insults, subsequently perfused transcardially with cold PBS and brains were removed. The cerebral infarct size was determined from 2,3,5-triphenyltetrazolium chloride (TTC)-stained, 2-mm-thick brain sections and the infarction areas were quantified using the iSolution full image analysis software (Image & Microscope Technology, Vancouver, Canada). To account for and eliminate the effects of swelling/edema, infarction volume was calculated by indirect measurement by summing the volumes of each section according to the following formula: contralateral hemisphere (mm^3^) - undamaged ipsilateral hemisphere (mm^3^). The edema volume was calculated as the damaged ipsilateral hemisphere (mm^3^) – the indirect infarct volume (mm^3^).

### Neurological score

Neurological deficit was scored in each mouse 24 h after ischemic insult in a blinded fashion by two investigators according to the following graded scoring system: 0 = no deficit; 1 = forelimb weakness and torso turning to the ipsilateral side when held by the tail; 2 = circling to the affected side; 3 = unable to bear weight on the affected side; and 4 = no spontaneous locomotor activity or barrel rolling [16].

### Wire-grip test

Vestibulo-motor function was assessed using a wire-grip test 24 h after cerebral ischemia in a blinded fashion by two investigators [17]. Briefly, mice were placed on a metal wire (45 cm long) suspended 45 cm above protective padding and allowed to traverse the wire for 60 s. The latency for which a mouse remained on the wire within a 60-s interval was measured, and the wire grip score was quantified using the following 5-point scale: unable to remain on the wire for 30 s = 0; failure to hold on to the wire with both sets of fore paws and hind paws together = 1; holding on to the wire with both forepaws and hind paws but not the tail = 2; holding on to the wire using the tail along with both forepaws and both hind paws = 3; moving along the wire on all four paws plus the tail = 4; a score of 4 points and also ambulating down one of the posts used to support the wire = 5. Tests were administered in triplicate and the average value was calculated for each mouse on each test day.

### Evans blue extravasation and water contents

BBB integrity was evaluated by Evans blue extravasation. Briefly, Evans blue (2 % in saline, 4 ml/kg; Sigma) was administered intravenously at the onset of ischemia. Mice were deeply anesthetized with thiopental sodium and then transcardially perfused with PBS to remove the intravascular dye 24 h after cerebral ischemia. Next, each hemisphere was weighed, homogenized in 2 ml of *N,N*-dimethylformamide (Sigma-Aldrich), incubated for 18 h at 55 °C, and then centrifuged (13,000 rpm for 10 min). The absorbance of the supernatant at 620 nm was then measured by spectrophotometry and the results were expressed as μg/g tissue calculated against a standard curve. The brain tissue water content was also measured by the wet and dry weight method 24 h after cerebral schemia. To accomplish this, the hemispheres were weighed before and after drying at 100 °C for 48 h, and the percentage of water content was calculated as 100 × (wet weight-dry weight)/wet weight.

### Western blotting

Mice were deeply anesthetized with thiopental sodium 24 h after the induction of ischemia, subsequently perfused transcardially with cold PBS and brain cortex was collected. Proteins were then isolated according to standard techniques, separated by 10 % sodium dodecyl sulfate-polyacrylamide gel electrophoresis, and transferred onto a nitrocellulose membrane (Amersham Biosciences, Piscataway, NJ). Next, immunoblot analysis was performed using anti-MMP-9 antibody (Millipore, Billerica, MA), as well as anti-ZO-1 and anti-occludin antibodies (Invitrogen, Camarillo, CA), followed by secondary antibody conjugated with horseradish peroxidase. The intensity of chemiluminescence was measured using an ImageQuant LAS 4000 apparatus (GE Healthcare Life Sciences, Uppsala, Sweden). The membrane was then reprobed with anti-β-actin antibody (Sigma-Aldrich) as an internal control.

## Data analyses

The data are expressed as mean ± standard error of mean (SEM). One-way analysis of variance (ANOVA) test was used to compare infarct volume, neurological score, wire grip test, Evans blue leakage, edema and brain water contents among the vehicle group, 1 mg/kg PPUS treated group or 3 mg/kg PPUS treated group. Unpaired *t*-test was used to compare MMP-9, ZO-1 and occluding expression between the control and the vehicle and between the vehicle and PPUS-treated groups. P < 0.05 was considered statistically significant. Statistical analysis was performed using SigmaPlot 11.2 (Systat Software Inc).

## Results

### PPUS attenuated brain infarction and improved functional outcome

**Fig. 1:**
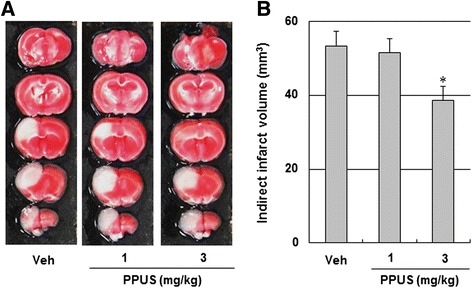
PPUS treatment reduced infarct volume after ischemic brain injury. (**a**) Representative photographs of coronal brain sections stained with 2,3,5-triphenyltetrazolium chloride in vehicle (Veh) and partially purified component of *Uncaria sinensis* (PPUS)-treated mice. Mice were intraperitoneally administered DMSO, 1 or 3 mg/kg PPUS 30 min before ischemic insult. White indicates the infarct area. (**b**) Quantification of the infarct volume 24 h after photothrombotic cortical ischemia. Data are expressed as means ± SEM (N = 5). * P < 0.05 when compared with the vehicle group (One-way ANOVA)

**Fig. 2:**
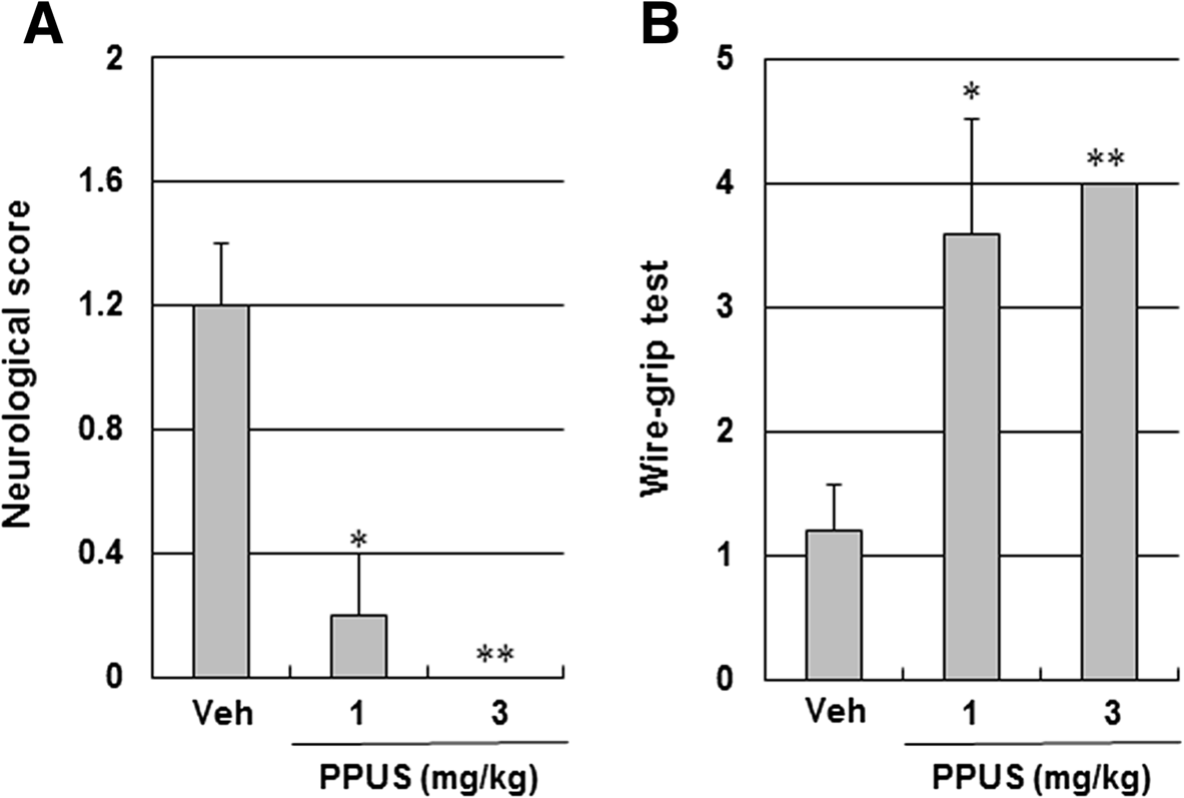
PPUS improved neurological and motor function after ischemic brain injury. Mice were intraperitoneally administered DMSO, 1 or 3 mg/kg partially purified component of *Uncaria sinensis* (PPUS) 30 min before ischemic insult. Quantification of (**a**) neurological deficit and (**b**) motor deficit were evaluated 24 h after photothrombotic cortical ischemia in a blinded fashion. Vestibule-motor function was assessed by a wire grip test. Data are expressed as means ± SEM (N = 5). * P < 0.05, ** P < 0.01 compared with the vehicle group (One-way ANOVA)

As shown in Fig. 1a, TTC staining revealed that infarct volume was significantly decreased at 24 h after ischemic brain injury in the 3 mg/kg PPUS-treated mice relative to the vehicle treated mice (38.6 ± 3.8 mm^3^ vs 53.4 ± 4.1 mm^3^, respectively, P < 0.05; Fig. 1b). Consistent with the infarct volume, ischemia-induced neurological deficits and vestibule-motor deficits, as determined from the neurological scores and the wire-grip test, respectively, were dose dependently attenuated in PPUS-treated mice 24 h after cortical infarction (Fig. 2). These findings suggested that PPUS improved tissue outcome and functional outcome in cerebral ischemic mice.

### PPUS protected the BBB

**Fig. 3:**
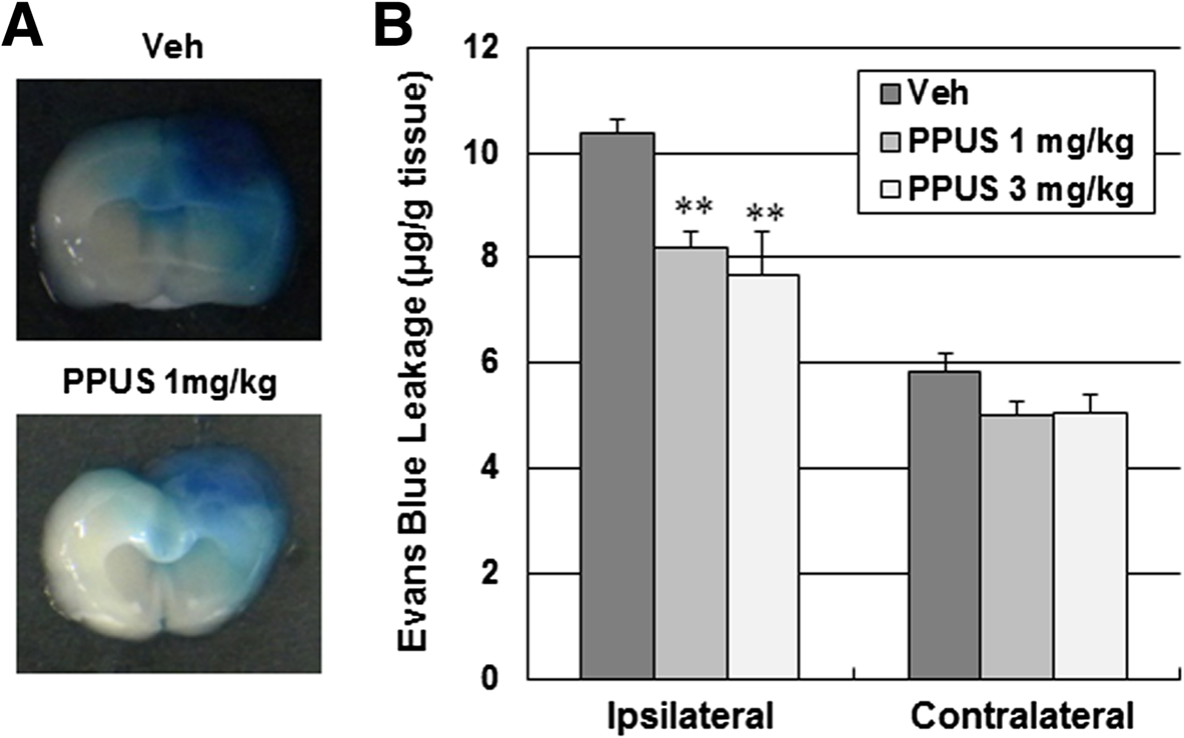
Evans blue extravasation was attenuated in PPUS-treated mice after ischemic brain injury. (**a**) Representative photographs of Evans blue leakage in coronal section of the brain in vehicle (Veh)- and partially purified component of *Uncaria sinensis* (PPUS)-treated mice 24 h after photothrombotic cortical ischemia. Mice were intraperitoneally administered DMSO or 1 mg/kg PPUS 30 min before ischemic insult. Blue area shows extravasated Evans blue, indicating BBB disruption. (**b**) Quantitative analysis of Evans blue leakage. Data are expressed as means ± SEM (N = 5). ** P < 0.01 compared with the vehicle group (One-way ANOVA)

**Fig. 4:**
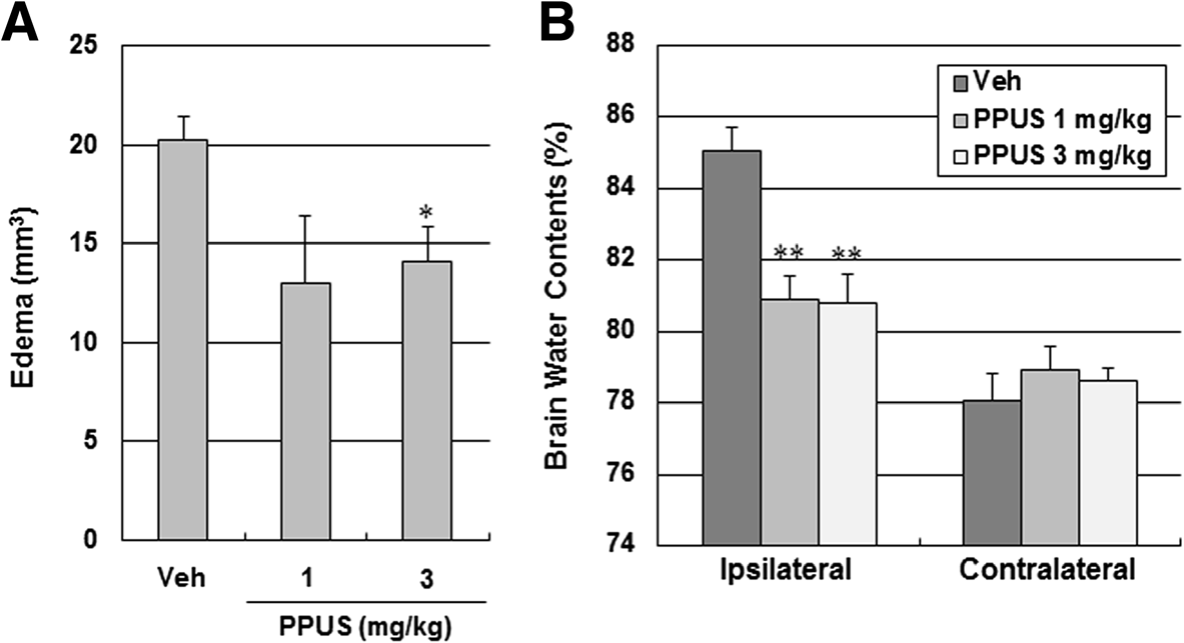
PPUS attenuated ischemic brain injury-induced edema. (**a**) Brain tissue edema volume (**a**) and water content (**b**) in vehicle (Veh)- and partially purified component of *Uncaria sinensis* (PPUS)-treated mice 24 h after photothrombotic cortical ischemia. Data are expressed as means ± SEM (N = 5). * P < 0.05, ** P < 0.01 compared with the vehicle group (One-way ANOVA)

To evaluate BBB permeability after ischemic brain injury, BBB leakage was measured using Evans blue extravasation. A marked increase in Evans blue content was observed in the ipsilateral hemisphere when compared with the contralateral side 24 h after ischemic brain injury. PPUS (1 and 3 mg/kg) markedly and dose dependently reduced Evans blue extravasation in the ipsilateral hemisphere after focal cerebral ischemia (Fig. 3), suggesting that PPUS protected the BBB. Brain edema, which was examined by TTC staining, was significantly decreased in response to treatment with 3 mg/kg PPUS (14.1 ± 1.7 mm^3^ vs 20.2 ± 1.2 mm^3^, respectively, P < 0.05; Fig. 4a). Concomitant with BBB disruption, the brain water content was increased notably in the ipsilateral hemisphere at 24 h; however, this increase was attenuated by PPUS (Fig. 4b).

### PPUS attenuated ischemic brain injury-induced tight junction degradation and MMP elevation

**Fig. 5:**
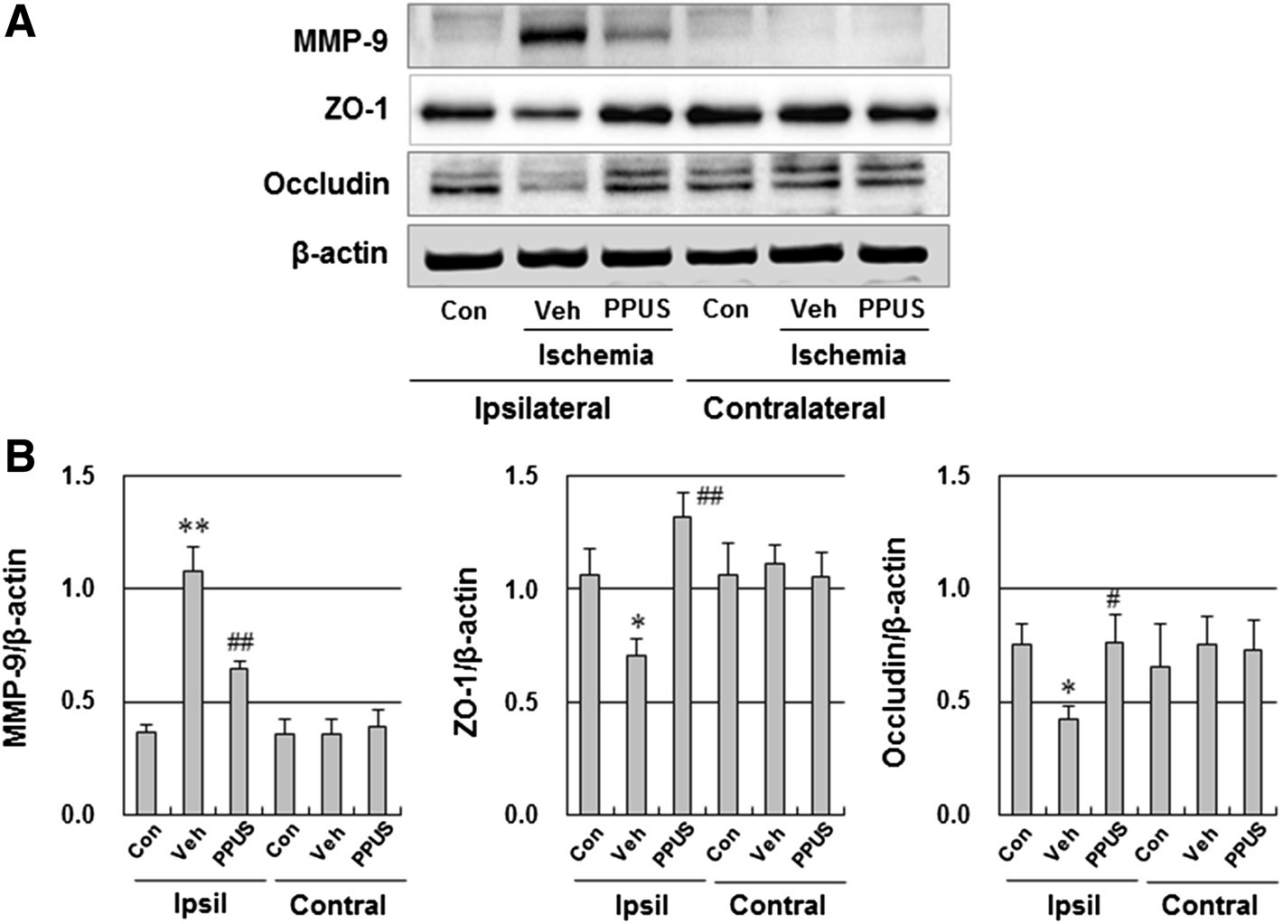
PPUS attenuated ischemic brain injury-induced tight junction degradation and MMP-9 elevation. (**a**) Representative western blots of MMP-9 and tight junction protein (ZO-1 and occludin) in brain tissues from DMSO- (Veh) and partially purified component of *Uncaria sinensis* (PPUS)-treated mice 24 h after ischemia. (**b**) Densitometric analysis of the western band. Mice were intraperitoneally administered DMSO or 3 mg/kg PPUS 30 min before ischemic insult. The control mice (Con) were without any ischemic injury. Zonula occludens-1 (ZO-1), occludin and MMP-9 protein levels were analyzed in both ischemic cortex (ipsilateral, Ipsil) and nonischemic cortex (contralateral, Contral) of the brain by Western blotting. PPUS restored ZO-1 and occludin downregulation and suppressed MMP-9 overexpression. Data are expressed as means ± SEM (N = 6). * P < 0.05, ** P < 0.01 compared with the control group; ^#^ P < 0.05, ^##^ P < 0.01 compared with the vehicle group (unpaired *t*-test)

To further investigate the effects of PPUS on tight junction proteins and MMP involved in BBB integrity, we measured ZO-1, occludin and MMP-9 by Western blotting. ZO-1 and occludin protein levels were significantly decreased and MMP-9 protein levels were remarkably upregulated in the ischemic cortex (Fig. 5). Additionally, the ischemic brain injury-induced tight junction protein downregulation and MMP-9 upregulation were prevented by PPUS treatment. However, ZO-1, occludin and MMP-9 protein levels were essentially unaffected by photothrombotic cortical ischemia in the contralateral hemisphere.

## Discussion

PPUS significantly reduced infarct volume and improved neurological and motor outcome after permanent focal cerebral ischemia. This protective effect of PPUS against brain ischemic injury involves its ability to attenuate BBB disruption and brain edema, which may involve suppression of ischemic brain injury-induced downregulation of ZO-1 and occludin and overexpression of MMP-9. Collectively, these results show that PPUS can efficiently prevent ischemic brain injury.

The hook and stems of US considered the active ingredient in Choto-san, which is used in the treatment of cardiovascular disease and various nervous-related symptoms [8]. The beneficial effects of Choto-san on vascular dementia were demonstrated in a double-blind, placebo-controlled study [18]. Additionally, it was reported that the oral administration of Choto-san prevented the occurrence of stroke and prolonged the life span of stroke-prone spontaneously hypertensive rats [19]. A number of in vitro and in vivo studies have investigated the beneficial protective effects of US and its mechanism of action. These studies have demonstrated that US had neuroprotective effects against glutamate-induced neuronal death in cultured cerebellar granule cells through the inhibition of Ca^2+^ influx [9, 10]. In other studies, US prevented delayed neuronal death of hippocampal neuronal cells in a transient forebrain ischemia gerbil model by reducing oxidative damage [11, 12]. Recently, our studies revealed that US had neuroprotective and cerebrovascular protective effects against ischemic brain damage. US exerted cerebrovascular protective action through an eNOS-dependent mechanism in cerebral ischemic mice and exhibited anti-apoptotic properties against glutamate-induced neurotoxicity in primary cultured cortical neurons [13, 14]. Despite the multiple pharmacological properties of US, including Ca^2+^ channel blocking, anti-oxidant and anti-apoptotic effects, together with its cerebrovascular protective effect, the effects of US on BBB breakage after focal cerebral ischemia has not been thoroughly investigated.

Cerebral microvessels possess barrier structures comprising tight junctions that are critical for maintenance of the homeostasis of the neural microenvironment [4]. BBB disruption is a critical event in the progression of ischemic stroke [5]. The loss of BBB integrity allows intravascular proteins and fluid to penetrate into the cerebral parenchymal extracellular space, leading to vasogenic edema formation and further brain damage. Accordingly, protecting the BBB and maintaining its integrity might be a beneficial method of alleviating brain damage. In this study, PPUS decreased brain tissue edema volume and water content after focal cerebral ischemia. In addition, Evans blue extravasation was attenuated in PPUS-treated mice after ischemic brain injury. These findings suggest that the preventive effect of PPUS in ischemic brain injury is involved in the reduction of brain edema.

The molecular mechanisms underlying ischemic brain injury-induced BBB opening have been well defined. Among various components of the BBB, the tight junction protein (ZO-1 and occludin) and MMP-9 are two of the most widely studied, and both are critical for maintaining the BBB structural integrity and permeability [4, 5, 20]. Degradation of the cerebrovascular ZO-1 and claudin as well as the MMP has been shown to be highly correlated with the dynamic process of BBB disruption after cerebral ischemia [6, 7]. MMPs mediate BBB disruption and vasogenic edema after cerebral ischemia by degrading the extracellular matrix, basal lamina proteins, and tight junctions around the BBB. Among MMPs, MMP-9 has been most intensively studied for its involvement in BBB disruption after stroke [7]. Severe BBB disruption is associated with marked elevation of MMP-9 at 24 to 48 h after ischemia [21, 22]. Furthermore, US was reported to have MMP-9 inhibitory ability in human aortic smooth muscle cells [23]. Therefore, it is possible that PPUS downregulates ischemic brain injury-induced MMP-9 elevation. Tight junction proteins are essential components of the BBB and substrates of MMPs. It has been reported that MMP-9 knockout mice are resistant to BBB disruption induced by focal cerebral ischemia and that this protection is mediated by reduced degradation of ZO-1 [24]. Similar findings were also reported using MMP pharmacological inhibitors [7]. Accordingly, we observed robust upregulation of MMP-9 in the focal ischemic brain, which was significantly reversed by PPUS. In addition, the administration of PPUS significantly reduced brain edema and rescued ischemia-induced ZO-1 and occludin disruption, suggesting that PPUS regulates BBB integrity through tight junction proteins. Taken together, our results suggest that MMP-9 is a key protease interfering with BBB integrity, and the ability of PPUS to block MMP-9 elevation, as well as ZO-1 and occludin downregulation is involved in BBB protection by PPUS.

## Conclusions

The BBB is one most important target of PPUS when preventing ischemic stroke. PPUS exerts preventive effects against the structure and function of brain damage under ischemic conditions. Specifically, PPUS maintains BBB integrity by blocking MMP-9 elevation and promoting the expression of ZO-1 and occludin. This and previously revealed beneficial effects of US on neuronal and cerebrovascular systems strongly point to their potential use for prevention of the development of ischemic brain injury.

## Abbreviations

PPUS: Partially purified components of *Uncaria sinensis*
BBB: Blood–brain barrier
MMP-9: Matrix metalloproteinase-9
